# Muscle Synergy Alteration of Human During Walking With Lower Limb Exoskeleton

**DOI:** 10.3389/fnins.2018.01050

**Published:** 2019-01-29

**Authors:** Zhan Li, Huxian Liu, Ziguang Yin, Kejia Chen

**Affiliations:** School of Automation Engineering, University of Electronic Science and Technology of China, Chengdu, China

**Keywords:** muscle, synergy, walking, exoskeleton, human

## Abstract

Muscle synergy reflects inherent coordination patterns of muscle groups as the human body finishes required movements. It may be still unknown whether the original muscle synergy of subjects may alter or not when exoskeletons are put on during their normal walking activities. This paper reports experimental results and presents analysis on muscle synergy from 17 able-bodied subjects with and without lower-limb exoskeletons when they performed normal walking tasks. The electromyography (EMG) signals of the tibialis anterior (TA), soleus (SOL), lateral gastrocnemius (GAS), vastus medialis oblique (VMO), vastus lateralis oblique (VLO), biceps femoris (BICE), semitendinosus (SEMI), and rectus femoris (RECT) muscles were extracted to obtain the muscle synergy. The quantitative results show that, when the subjects wore exoskeletons to walk normally, their mean muscle synergy changed from when they walked without exoskeletons. When the subjects walked with and without exoskeletons, statistically significant differences on sub-patterns of the muscles' synergies between the corresponding two groups could be found.

## 1. Introduction

Combinational movements of multiple joints essentially result in human body motion. Joints are actuated by associated muscle groups which are synergistically manipulated by the neural signals from the central nervous system (CNS). As we may know, muscle groups possess high redundancy to achieve potential flexibility for joints, but they still follow limited coordination manners to finish various motor tasks. Such inherent coordination manners of muscles (i.e., muscle synergy) can be perceived as natural and optimal in CNS level. In the past decades, many researchers mainly focused on analyzing muscle synergy of people doing motor learning and locomotion tasks without using wearable assistive robots. In a pioneering work, d'Avella et al. pointed out that a set of muscle synergies basically constructs motor behaviors and that they are highly related to kinematics (d'Avella et al., 2003; Tresch et al., 2006). Chvatal et al. analyzed common muscle synergies for control of center of mass (CoM) for stepping and non-stepping postural responses, revealing that for some similar motor tasks the subject may share common muscle synergies (Chvatal et al., 2011). Zwaan et al. applied muscle synergies to investigate selective motor control in cerebral palsy in gait, supporting the sensitive nature of EMG to represent an aberrant motor control (Zwaan et al., 2012). Fautrelle et al. investigated the latencies of muscular activities and the way they are correlated between certain muscles to stress the muscular synergies involved in movement and, in their study, they suggested the CNS reprograms a new synergy after the target jumps in order to correct the ongoing reaching movement (Fautrelle et al., 2010). Wojtara et al. proposed a synergy-based stability index during maintaining lateral balance, and this work considers the temporary muscle synergies in postural reflex and automatic response (Wojtara et al., 2014). Wang et al. analyzed muscle synergies facing a step made with obstacles in elderly people and revealed a decreased ability to use multiple-mode synergies following a predictable perturbation (Wang et al., 2015). Li et al. analyzed muscle synergy in the crus for examining its correlation with plantar/dorsiflexion in the ankle joint (Li et al., 2015).

The exoskeleton system is one kind of rehabilitation robots which enables the human knee joint to do daily movement training (Gui et al., 2017), such as being an active orthoses for injured pilots to correct abnormal gait. To assess wearing/training effects on subjects who use rehabilitation robots for daily movement (Zhang et al., 2017a), measurement and evaluation of their muscle activities is important in addition to analysis of kinematics/kinetics. Moreover, inducting muscle coordination information into exoskeletons for assistance of normal walking may be beneficial to human-in-the-loop optimization of energy flows (Zhang et al., 2017b). For instance, Alibeji et al. integrated muscle synergy into the control of hybrid walking neuroprosthesis (Alibeji et al., 2015). Estimating lower leg muscle activity can be achieved from distal bio-signals around the ankles (Isezaki et al., 2017). Upper limb exoskeletons have taken the muscle synergy effect into account in their design process (Burns et al., 2017), and muscle recruitment and coordination information is utilized to optimize the control of ankle exoskeletons (Steele et al., 2017). However, there is still a lack of research on investigation and evaluation of muscle synergy for subjects who perform normal walking while wearing lower-limb exoskeletons. It is important to observe how their muscle synergies would alter when equipping such wearable robots to assist walking. Such muscle co-contraction alteration is worthy of investigation to assess potential side effects for muscles from exoskeletons, especially for subjects with long-term use of exoskeletons, and their muscle synergies might be gradually transformed due to plasticity. Thus, analysis of muscle synergies with lower-limb exoskeletons may be important and beneficial to the design of novel exoskeleton systems toward achieving more natural muscle co-contraction patterns for locomotion and in daily life.

This paper aims to investigate such potential alteration effects of muscle synergies in able-bodied subjects when wearing lower limb exoskeleton systems in performing normal walking tasks, continuing our preliminary work on muscle synergy analysis for quiet standing in healthy subjects (Li et al., 2016). This work tries to investigate how muscle synergy patterns can be affected by lower-limb exoskeleton systems to assist normal dynamic walking. To the best of our knowledge, there is little work focusing specifically on this topic. We would like to present the muscle synergy alteration details with contrasted co-contraction sub-patterns of muscle groups among able-bodied people before and after equipping lower-limb exoskeletons to walk. EMG signals of eight muscles in the lower extremities of both legs of 17 healthy subjects were acquired and processed during the subjects' normal walking with and without wearing lower-limb exoskeletons, and the muscle synergy on a single leg is extracted to present the muscle coordination patterns in different reduced dimensions. In the following statistical results on the muscle synergy of the 17 subjects, it can be observed that the average muscle synergy of the subjects changed when the subjects wear exoskeletons to do normal walking. Statistical results indicate the level of significant difference that muscle synergy alteration phenomena can be reached before and after wearing exoskeletons.

## 2. Materials and Methods

In this section, EMG signals of eight muscles of 17 subjects are acquired and analyzed to examine muscle synergies during walking in case of wearing exoskeletons and without exoskeletons.

### 2.1. Experiment Setup

Seventeen able-bodied subjects (16 male and 1 female, 22.88 ± 1.32 years old, 173.65 ± 5.22 cm height, and 54.59 ± 5.21 kg weight) participated in this study upon their consent. The experiments were exempted from IRB approval and followed the institutional guidelines of the University of Electronic Science and Technology of China, and all the experiment operations were in accordance with the Declaration of Helsinki. None of them had ever suffered neuromuscular disorders in their lower limbs. They were all instructed to utilize the lower limb exoskeletons to perform normal walking tasks. The lower limb exoskeleton system used in the experiment was developed by the University of Electronic Science and Technology of China. The lower limb exoskeleton system has four active degrees of freedom (flexion/extension) of motion in hip and knee joints, and its ankle joints have two passive degrees of freedom of motion (dorsi- and plantar flexion). The subjects are required to use crutches to maintain balance during locomotion for safety.

**Figure 1:**
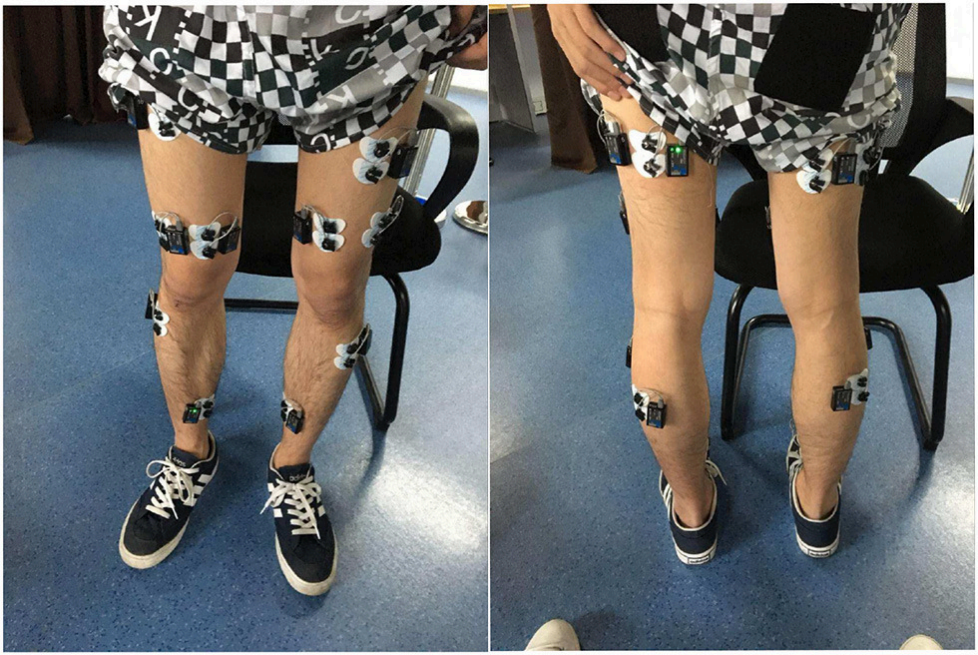
EMG electrode locations on the lower limb of one able-bodied subject.

Surface EMG signals were acquired by a commercial EMG acquisition system (TeleMyo DTS System, Noraxon Ltd., Scottsdale, Arizona, United States). The placement of the EMG acquisition pods/electrodes on anterior and posterior sides of lower limbs is shown in Figure 1. Eight muscles around the knee, ankle, and hip joints were selected to be tested: the tibialis anterior (TA), soleus (SOL), lateral gastrocnemius (GAS), vastus medialis oblique (VMO), vastus lateralis oblique (VLO), biceps femoris (BICE), semitendinosus (SEMI), and rectus femoris (RECT) muscles in the lower limbs. Eight channels of bipolar differential amplifier were carefully placed on these muscles on each leg according to both the anatomy and joint flexion/rotation experience. The EMG electrodes of each channel were positioned at the muscle belly along the muscle fiber direction with the reference electrode orthogonal to the midline of the active electrodes according to the recommendation of Noraxon. The skin underneath the electrodes was cleaned to reduce the resistance between the skin and the electrodes. The EMG signals were amplified and sampled at 1,500 Hz. The acquired raw EMG signals were rectified and low-pass filtered with a 4th-order Butterworth filter under a 15 Hz cutoff frequency.

### 2.2. Experimental Protocol

**Figure 2:**
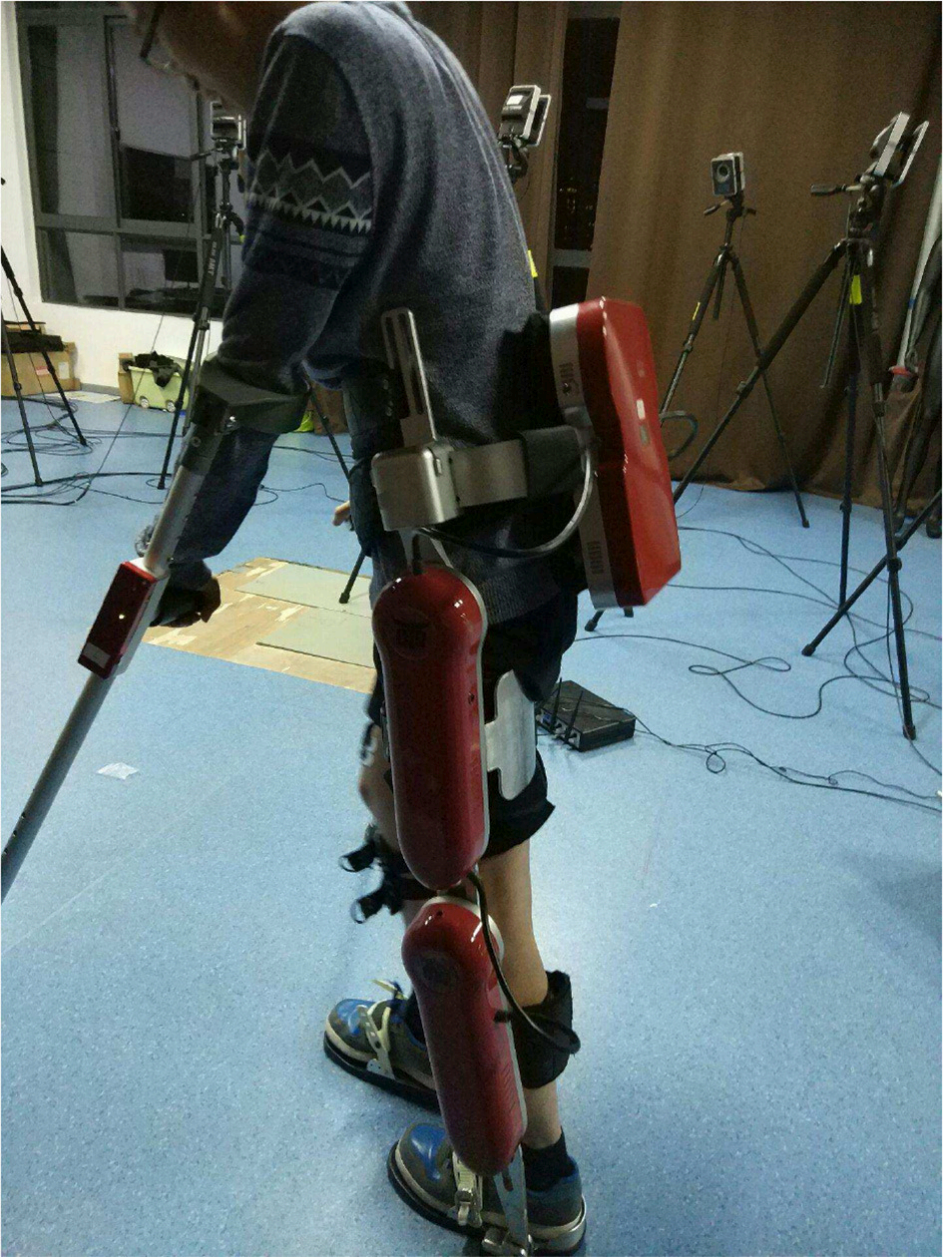
One subject is wearing the lower-limb exoskeleton system and doing a normal walking task. (Consent was obtained from the individual for the publication of this image).

In the experiment, all 17 subjects were individually instructed to perform two types of normal walking tasks, i.e., the first test session for each subject was to let him/her wear the exoskeleton to walk, and the second test session for each subject was to let him/her walk without wearing the exoskeleton. These two sessions are independent and separate. In the first session for normal walking, every subject was told to walk 10 m at a rate of 1 step per second. They stopped for a short time and repeated the same walking rhythm as they had just finished. All the subjects repeated this normal walking trial 4 times. After they completed the first session, they rested for a while and then wore the exoskeletons. In the second session for walking with exoskeletons, each subject was told to walk 5 m without speed restriction, and they repeated the walking tasks with the exoskeletons 4 times. Figure 2 shows one subject wearing the exoskeleton and walking in the experiment.

### 2.3. Muscle Synergy Extraction

After all the EMG signals of all eight channels on the 17 subjects were acquired and filtered, we extracted the muscle synergies in their right legs as the following procedures. First, we construct the following multiple-channel EMG signal matrix *U* acquired for each individual

(1) $$\begin{array}{r} U=\left[\begin{array}{c} U_{\text{TA}}\\ U_{\text{SOL}}\\ U_{\text{GAS}}\\ U_{\text{VMO}}\\ U_{\text{VLO}}\\ U_{\text{BICE}}\\ U_{\text{SEMI}}\\ U_{\text{RECT}} \end{array}\right]=\left[\begin{array}{cccc} U_{\text{TA}}\left(1\right) & U_{\text{TA}}\left(2\right) & \cdots & U_{\text{TA}}\left(N\right)\\ U_{\text{SOL}}\left(1\right) & U_{\text{SOL}}\left(2\right) & \cdots & U_{\text{SOL}}\left(N\right)\\ U_{\text{GAS}}\left(1\right) & U_{\text{GAS}}\left(2\right) & \cdots & U_{\text{GAS}}\left(N\right)\\ U_{\text{VMO}}\left(1\right) & U_{\text{VMO}}\left(2\right) & \cdots & U_{\text{VMO}}\left(N\right)\\ U_{\text{VLO}}\left(1\right) & U_{\text{VLO}}\left(2\right) & \cdots & U_{\text{VLO}}\left(N\right)\\ U_{\text{BICE}}\left(1\right) & U_{\text{BICE}}\left(2\right) & \cdots & U_{\text{BICE}}\left(N\right)\\ U_{\text{SEMI}}\left(1\right) & U_{\text{SEMI}}\left(2\right) & \cdots & U_{\text{SEMI}}\left(N\right)\\ U_{\text{RECT}}\left(1\right) & U_{\text{RECT}}\left(2\right) & \cdots & U_{\text{RECT}}\left(N\right) \end{array}\right] \end{array}$$

where *U*_*j*_ (*j*∈{TA, SOL, GAS, VMO, VLO, BICE, SEMI, RECT}) denotes the EMG time sequence of each type of muscle in the right leg with total *N* samplings. The non-negative matrix factorization (NMF) method is applied (Tresch et al., 2006) to decompose *U*∈*R*^8 × *N*^ as

$$\begin{array}{l} U=WH \end{array}$$

where *W*∈*R*^8 × *k*^ denotes the muscle synergy ratio matrix and *H*∈*R*^*k*×*N*^ denotes the extracted synergy intensity matrix (neural commands). The decomposition for updating entries *h*_*kl*_ and *w*_*jk*_ of *H* and *W* is conducted with the following iterative algorithm

$$\begin{array}{l} h_{kl}\leftarrow h_{kl}\frac{{\left[W^{T}U\right]}_{kl}}{{\left[W^{T}WH\right]}_{kl}}\\ w_{jk}\leftarrow w_{jk}\frac{{\left[UH^{T}\right]}_{jk}}{{\left[WHH^{T}\right]}_{jk}} \end{array}$$

The algorithm is performed by calling the “nnmf” function built in MATLAB R2016a, by minimizing the cost function (residual error) ||*U*−*WH*||_*F*_, where ||·||_*F*_ denotes Frobenius norm. The iterative method starts with random initial values for *W* and *H*. The entries of synergy matrix *W* in each of its columns come into being as the muscle co-contraction patterns with different choices of reduced dimension *k*, i.e., the vector combined by entries *w*_1*k*_, *w*_2*k*_, ⋯, *w*_8*k*_ denotes Synergy *k*. For example, in case of *k* = 3, there are three total types of synergy, i.e., the vector combined by entries *w*_11_, *w*_21_, ⋯, *w*_81_ represents Synergy 1, the vector combined by entries *w*_12_, *w*_22_, ⋯, *w*_82_ represents Synergy 2, and the vector combined by entries *w*_13_, *w*_23_, ⋯, *w*_83_ represents Synergy 3.

## 3. Results and Discussions

In this section, muscle synergies of the 17 able-bodied subjects were extracted by NMF with dimension *k* being reduced to 3, 4, and 6 from the acquired EMG signals, respectively. The muscle synergies of subjects who wear lower-limb exoskeletons for subjects are compared with those of subjects without wearing lower-limb exoskeletons. Analysis of variance (ANOVA) was used to evaluate the statistical significance between muscle synergies with an exoskeleton (i.e., *W*_with_) and those without an exoskeleton (i.e., *W*_without_). The *p*-value matrices were calculated. If *p* ≤ 0.05 holds between each synergy value *W*_with_ and *W*_without_ correspondingly, then the statistical significance of muscle synergy alteration can be seen.

### 3.1. Muscle Synergy With Extraction Dimension *k* = 3

**Figure 3:**
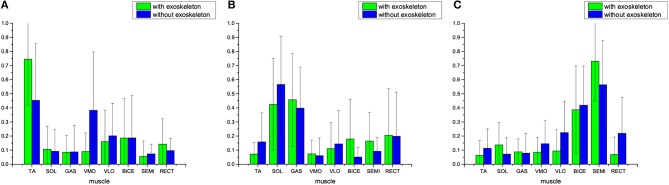
Average muscle synergies of the 17 subjects who walk with and without lower-limb exoskeletons. NMF is used to exact the muscle synergy with reduced dimension being *k* = 3. **(A)** Synergy 1, **(B)** Synergy 2, and **(C)** Synergy 3.

In this case, the reduced dimension in NMF is *k* = 3 for muscle synergy extraction from multiple-channel EMG signals, i.e., there are three synergy patterns: Synergy 1, Synergy 2, and Synergy 3. Figure 3 comparatively shows the average muscle synergies of the 17 subjects during their normal walking tasks with and without wearing lower limb exoskeletons. More specifically, to further show the statistical significance for the muscle synergy alteration effect, the *p*-values are shown in Table 1. From Figure 3, we can observe that the average muscle synergy patterns of the 17 subjects who wear lower-limb exoskeletons during normal walking are altered from those of the subjects who perform normal walking without exoskeletons. As seen from Synergy 1 in Figure 3A, we find that, when the subjects wear an exoskeleton for walking, their TA muscles exhibit a dominant role with little co-contraction effects from other muscles. For comparison, when the subjects walk without an exoskeleton, their TA muscles still keep the main contributed role, but different co-contraction patterns appear. As shown in Table 1, the differences between Synergy 1 with and without an exoskeleton mainly focus on TA and VMO muscles' contraction patterns are statistically significantly different, since their corresponding *p*-values are both <0.05. For Synergy 2 shown in Figure 3B, we can see the co-contraction patterns are quite different as well. When the subjects walk with and without exoskeletons, SOL and GAS muscles are always the main contributed ones. However, the *p* values for TA, SOL, BICE, and SEMI muscles are <0.05, which may indicate the co-contraction patterns from the two muscles are significantly altered. We observe Synergy 3 in Figure 3C and can find that BICE and SEMI muscles are the main contributions. The *p*-values for TA, SOL, VMO, VLO, SEMI, and RECT muscles are <0.05, and it indicates that wearing exoskeletons might change muscle co-contraction patterns.

**Table 1:** The *p*-values between muscle synergy $W_{\text{with}}\in R^{3\times 8}$ and $W_{\text{without}}\in R^{3\times 8}$, *p* ≤ 0.05 indicates significant difference in statistics.

** 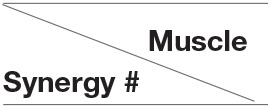 **	**TA**	**SOL**	**GAS**	**VMO**	**VLO**	**BICE**	**SEMI**	**RECT**
Synergy 1	**0.0000**	0.5986	0.9642	**0.0000**	0.3005	0.9916	0.2723	0.0587
Synergy 2	**0.0019**	**0.0157**	0.2615	0.5089	0.3696	**0.0003**	**0.0084**	0.9070
Synergy 3	**0.0199**	**0.0077**	0.6192	**0.0136**	**0.0001**	0.5407	**0.0015**	**0.0000**

*The bold value is < 0.05*.

### 3.2. Muscle Synergy With Extraction Dimension *k* = 4

**Figure 4:**
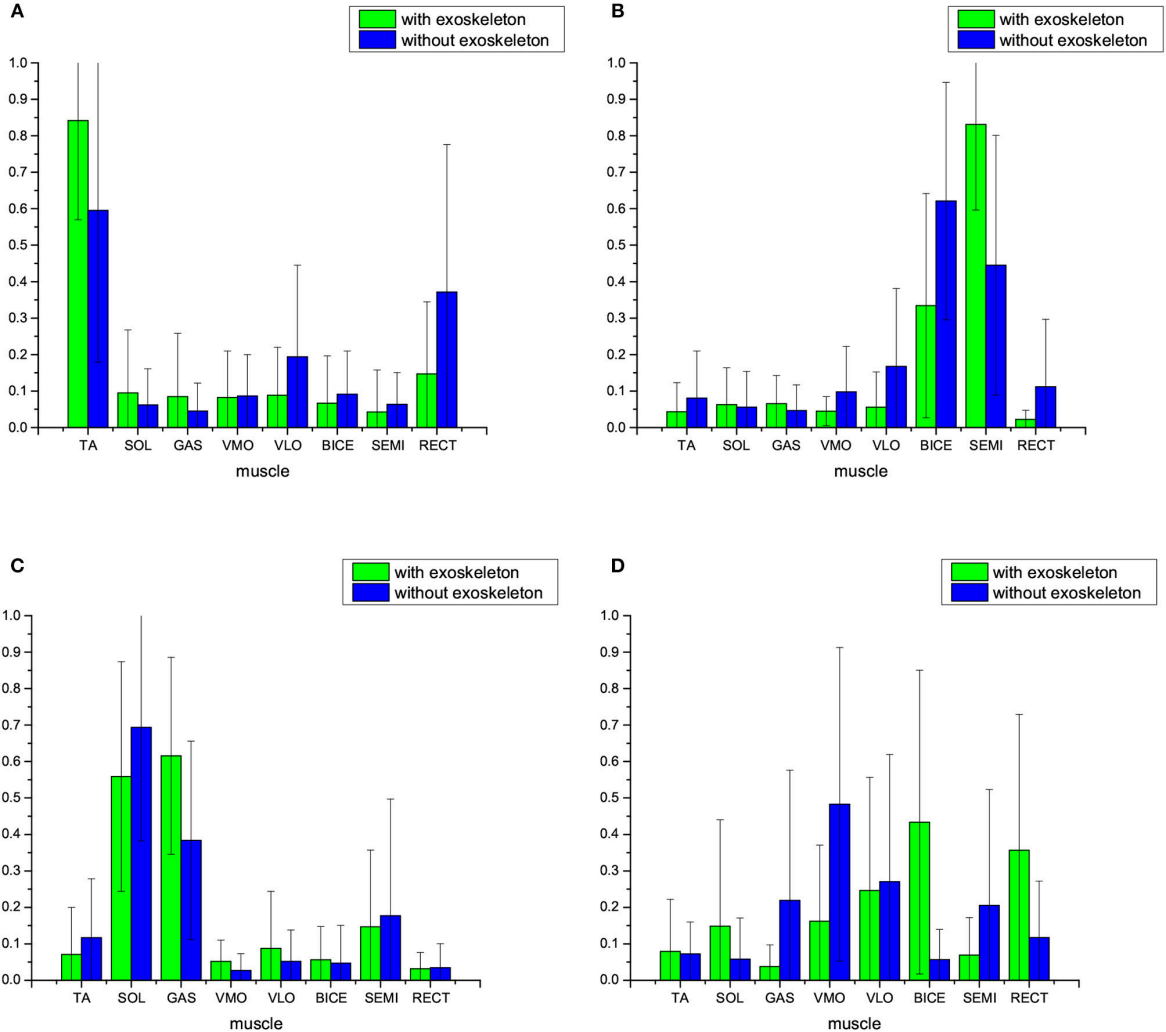
Average muscle synergies of the 17 subjects who walk with and without lower-limb exoskeletons. NMF is used to exact the muscle synergy, with the reduced dimension being *k* = 4. **(A)** Synergy 1, **(B)** Synergy 2, **(C)** Synergy 3, and **(D)** Synergy 4.

In this part of the results, muscle synergies were extracted by NMF with reduced dimension being *k* = 4, i.e., Synergies 1~4 are produced. Figure 4 shows the average muscle synergy of the 17 subjects with and without lower-limb exoskeletons to perform normal walking. Table 2 shows the statistical significance results for muscle synergy with and without an exoskeleton. From Figure 4 we can observe that, when the subjects wear exoskeletons to walk, the TA muscle is still the main contributing muscle and other muscles' co-contractions are almost non-existent for Synergy 1, BICE and SEMI muscles are the main contributing muscles for Synergy 2, the GAS muscle can be seen as the main contributor for Synergy 3, and BICE and RECT muscles play the dominant roles for Synergy 4. For comparison, Figure 4 also presents the mean average muscle synergy of the 17 subjects who do the same normal walking tasks without wearing lower-limb exoskeletons. From the synergy results without exoskeletons in Figure 4 we can see that, for Synergy 1, the TA muscle is the main contributor with co-contractions from VLO and RECT muscles; for Synergy 2, BICE and SEMI muscles are the main contributors; for Synergy 3, SOL and GAS are the main contributors to the movement; for Synergy 4, VMO becomes the main contributor. As reflected from the *p*-values in Table 2, we could conclude that the TA, VLO, and RECT muscles' synergies are changed in Synergy 1, the TA, VLO, BICE SEMI, and RECT muscles' synergies are changed in Synergy 2, SOL and GAS muscles' synergies are changed in Synergy 3, and SOL, GAS, VMO, BICE, SEMI, and RECT muscles' synergy are changed in Synergy 4. When examining muscle synergy extraction with dimension *k* = 4, muscle synergy alteration seems to occur more frequently.

**Table 2:** The *p*-values between muscle synergy $W_{\text{with}}\in R^{4\times 8}$ and $W_{\text{without}}\in R^{4\times 8}$, *p* ≤ 0.05 indicates significant difference in statistics.

** 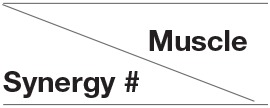 **	**TA**	**SOL**	**GAS**	**VMO**	**VLO**	**BICE**	**SEMI**	**RECT**
Synergy 1	**0.0001**	0.1714	0.0905	0.8523	**0.0028**	0.2488	0.2485	**0.0001**
Synergy 2	**0.0475**	0.6851	0.1254	**0.0012**	**0.0001**	**0.0000**	**0.0000**	**0.0001**
Synergy 3	0.0687	**0.0135**	**0.0000**	0.0069	0.1049	0.6015	0.5270	0.7691
Synergy 4	0.7463	**0.0187**	**0.0001**	**0.0000**	0.6708	**0.0000**	**0.0011**	**0.0000**

*The bold value is < 0.05*.

### 3.3. Muscle Synergy With Extraction Dimension *k* = 6

**Figure 5:**
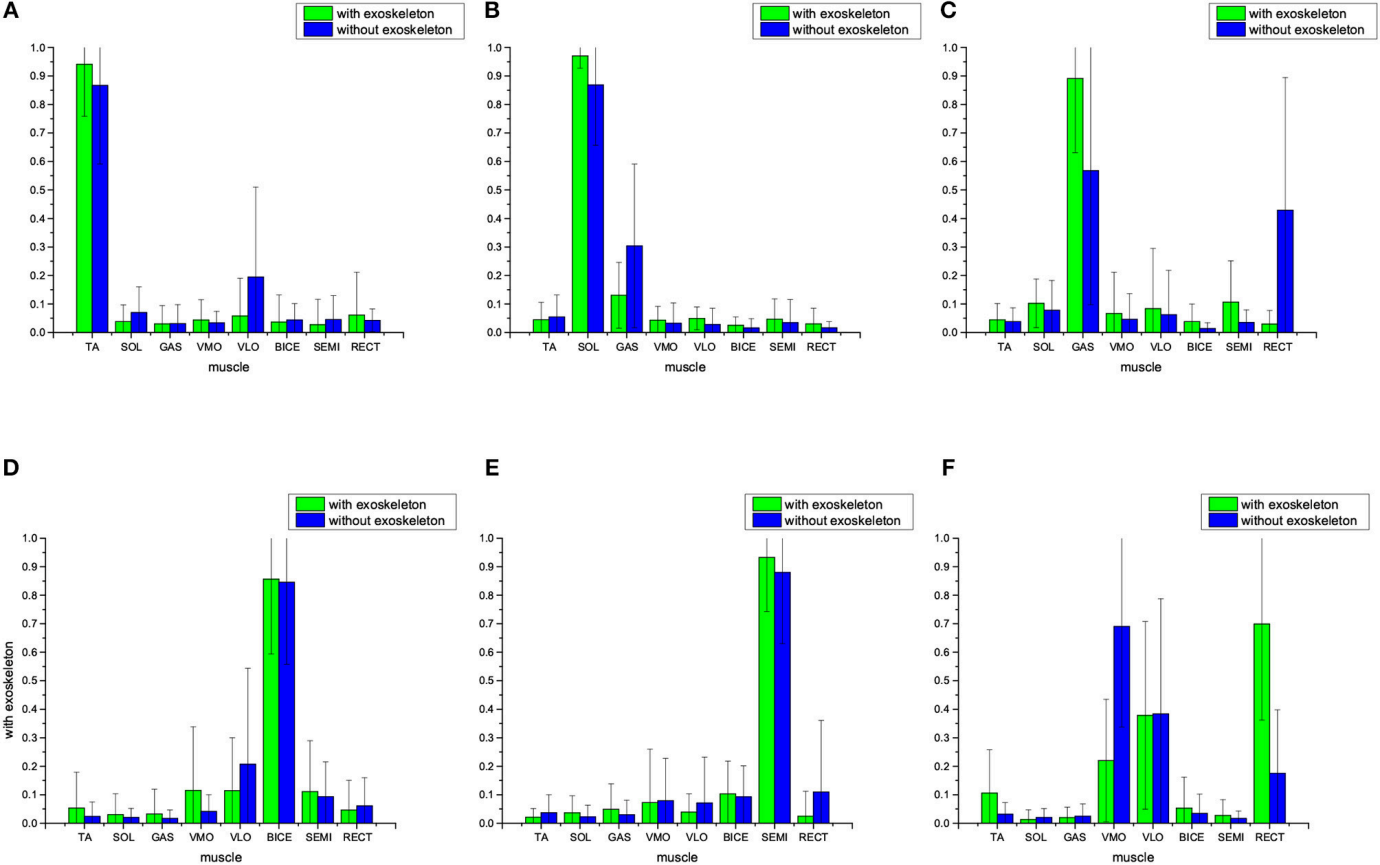
Average muscle synergy of the 17 subjects who walked with and without lower-limb exoskeletons. NMF was used to exact the muscle synergy with reduced dimension being *k* = 6. **(A)** Synergy 1, **(B)** Synergy 2, **(C)** Synergy 3, **(D)** Synergy 4, **(E)** Synergy 5, and **(F)** Synergy 6.

NMF was applied with reduced dimension *k* = 6 for muscle synergy extraction in the subsection. Figure 5 shows the average muscle synergy of the 17 subjects who were with and without exoskeletons for their normal walking tasks. For comparison, Figure 5 shows the average muscle synergy pattern of the 17 subjects who finished the same normal walking tasks without lower limb exoskeletons. Table 3 shows the *p*-values which represent statistical significance results for muscle synergy with and without exoskeleton. From Figure 5, we can observe that, when the subjects wear the exoskeleton for walking, the synergies seem altered as compared with those in case of walking without wearing exoskeletons. When the subjects walk with exoskeletons, for Synergy 1, TA muscle still keeps the role of the dominant contributor to the movement with less co-contractions from other muscles, and such similar phenomenon also appears when the reduced dimension becomes *k* = 3 or *k* = 4; for Synergy 2, SOL muscle is the main contributor muscle; for Synergy 3, GAS muscle acts as the main contributed muscle more distinctly; for Synergy 4, BICE muscle is still the main contributor; for Synergy 5, SEMI is still the main contributor; for Synergy 6, RECT seems to be the main contributor muscle instead of VLO muscle. From the statistical significance results in Table 3, the muscle synergy alteration effect also appears in all 6 synergy patterns. In Synergy 1, the SOL and VLO muscles' synergies are significantly different; in Synergy 2, the SOL, GAS, VLO, and BICE muscles' synergies are significantly different; in Synergy 3, the GAS, BICE, SEMI, and RECT muscles' synergies are significantly different; and in Synergy 4, only the VMO muscle's synergy is significantly different; in Synergy 5, only the RECT muscle's synergy is significantly different and in Synergy 6, the TA, VMO, and RECT muscles' synergies are significant different.

**Table 3:** The *p*-values between muscle synergy $W_{\text{with}}\in R^{6\times 8}$ and $W_{\text{without}}\in R^{6\times 8}$, *p* ≤ 0.05 indicates significant difference in statistics.

** 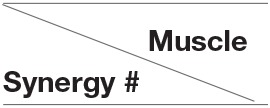 **	**TA**	**SOL**	**GAS**	**VMO**	**VLO**	**BICE**	**SEMI**	**RECT**
Synergy 1	0.0673	**0.0187**	0.9204	0.3339	**0.0014**	0.5970	0.2218	0.3327
Synergy 2	0.4091	**0.0002**	**0.0000**	0.3087	**0.0131**	**0.1011**	0.3680	0.0587
Synergy 3	0.5264	0.1450	**0.0000**	0.3174	0.4987	**0.0017**	**0.0002**	**0.0000**
Synergy 4	0.0754	0.3029	0.1710	**0.0094**	0.0519	0.8273	0.5002	0.4074
Synergy 5	0.0722	0.1115	0.1294	0.8333	0.1352	0.5765	0.1682	**0.0094**
Synergy 6	**0.0002**	0.2191	0.4610	**0.0000**	0.9300	0.2477	0.1770	**0.0000**

*The bold value is < 0.05*.

### 3.4. Discussion

From the aforementioned muscle synergy results with and without exoskeletons in different extraction dimensions *k* = 3, 4, and 6, we find that, when the subjects wore exoskeleton for normal walking, the corresponding muscle co-contraction patterns could be altered. Statistically significant results further demonstrate that such alteration effects may concentrate on some muscles. As seen from the *p* value results in Tables 1–3, two groups of muscle synergies of the eight present significant statistical difference (i.e., *p* ≤ 0.05) in different levels of extent, and all the sub-patterns from these muscle synergies show at least one muscle's contribution is significantly different. When the extraction dimension is chosen as *k* = 3, the TA muscle's synergies with and without exoskeletons show significant difference as the *p*-values in the three synergy patterns are < 0.05. The SOL, VMO, and SEMI muscles' synergies with and without exoskeletons show significant difference as well. Wearing an exoskeleton while walking does not affect only the GAS muscle's contribution. When the extraction dimension is set as *k* = 4, all eight muscles' synergies with and without an exoskeleton present significant difference, with *p* ≤ 0.05 appearing twice or more in Table 2. When we extract muscle synergy with dimension *k* = 6, all of the eight muscles' synergies with and without exoskeleton have chances to show significant difference. According to our previous work (Li et al., 2015), we can observe that some sub-patterns of muscle synergy have high correlations with joint movement (e.g., flexion or extension). This muscle synergy alteration indicates that human joint torque may be changed due to the involvement of exoskeleton joint torque. Thus, accurate measurement of the participation of assisted robots (e.g., robot torque) and human spontaneously-generated motion (e.g., human torque), together with clear distinction between them, can provide more insightful investigations on the cause of such significant differences.

From another point of view, utilizing lower-limb exoskeletons may change original patterns of muscle co-contractions in subjects during their normal walking activities. This may be not beneficial to the exercise of muscles of subjects who use exoskeletons frequently, since the natural and comfortable muscle synergy can be broken. In order to improve the co-contraction situations when the subjects wear exoskeletons to walk, it is necessary to design a muscle-contraction-primitive controller for exoskeletons instead of purely providing motion compensation by actuators. The users usually give feedback that they may feel uncomfortable and unnatural when they wear exoskeletons for walking. Based on observations of muscle synergy results, one can conclude the reason may lie in the fact that the original natural muscle synergies are altered to artificial ones when subjects use the exoskeletons, and the natural muscle's coordination patterns may be changed manually and compulsively during the process of subjects adapting themselves to exoskeletons. In order to make subjects' muscle synergies with assisted exoskeletons more similar to those without exoskeleton equipped, the following generalized procedure can help to improve the design of exoskeletons toward more natural motion assistance. First, through the aforementioned statistical significance results we can observe which specific muscle's contribution to movement is changed; secondly, by utilizing correlations between muscle synergy patterns and human joint torques in different degrees of freedom, we could improve the design to make the corresponding degree of freedom of the exoskeleton joint possess actuation; next, the level of actuation is adjusted according to exoskeleton dynamics with feedback from kinematics and EMG. Some works try to use EMG signals to control the exoskeleton by considering EMG as some sort of interpretation from human intentions (Kinnaird and Ferris, 2009; Kiguchi and Hayashi, 2012; Lenzi et al., 2012). In this case, the subjects' motion intention explicitly drives the contraction of one or more muscle groups to change EMG signals instead of subconsciously invoking inherent muscle coordination patterns. Involvement of synergistic information may be propitious to produce more natural motion for wearable exoskeleton devices (Hassan et al., 2018; Liu et al., 2018).

In the walking tasks not assisted by exoskeletons, the subjects perform their movement without crutches, as they can keep balance naturally as their daily walking movement. When the subjects wear exoskeletons to move, the crutches are used to maintain balance for safety reasons, with the hip and knee motion assisted by exoskeletons. The actuation of the human-exoskeleton hybrid system is composed of human muscle groups and robot motors. It is still a challenge to measure separately the torque from subjects and the torque from exoskeletons and how these torque values distribute and combine to cooperatively fulfill optimized motion in walking. This work presents that muscle synergy alteration effects appear when able-bodied subjects wear exoskeletons to walk rather than at the actuation level. Future development of advanced measurement technology on the torques of the ankle, knee, and hip joints synchronously together with EMG signals on their associated muscles may promote physiological interpretations of the reduced dimension number *k* for muscle synergy pattern extraction, as following the way of our previous work (Li et al., 2015). In case torque measurement of multiple joints in the lower extremities is lacking, utilization of EMG signals to analyze the muscle synergy might be a feasible manner to investigate the subjects' muscles' adaption effects to wearable robots.

## 4. Conclusions

This paper aims to investigate potential alteration effects of muscle synergies of able-bodied subjects after wearing lower limb exoskeleton systems when performing normal walking tasks. EMG signals from eight muscles in the lower extremities of one leg on 17 healthy subjects are used and processed to extract muscle synergies for these subjects to perform normal walking with and without wearing exoskeletons. According to the muscle synergy results of the 17 subjects, we see that patterns of average muscle synergy are changed obviously after the subjects wear exoskeletons. Statistical analysis further shows significant differences among sub-patterns in muscle synergies with and without exoskeletons, indicating that such alteration phenomena evidently exist.

## Author Contributions

ZL conceived of the study and designed the experiments. ZY designed the system. HL and KC conducted the experiment. HL and analyzed the data. All the authors drafted the manuscript.

### Conflict of Interest Statement

The authors declare that the research was conducted in the absence of any commercial or financial relationships that could be construed as a potential conflict of interest.
